# Urine sTREM-1 may be a valuable biomarker in diagnosis and prognosis of sepsis-associated acute kidney injury

**DOI:** 10.1186/s13054-015-0998-2

**Published:** 2015-07-14

**Authors:** Longxiang Su, Lixin Xie, Dawei Liu

**Affiliations:** Department of Critical Care Medicine, Peking Union Medical College Hospital, Peking Union Medical College & Chinese Academy of Medical Sciences, 1st Shuaifuyuan, Dongcheng District, Beijing 100730 China; Department of Pulmonary & Critical Care Medicine, Chinese PLA General Hospital, 28th Fuxing Road, Haidian District, Beijing 100853 China

## Abstract

Urine soluble triggering receptor expressed on myeloid cells-1 (sTREM-1) has been reported in sepsis diagnosis and prediction of sepsis-associated acute kidney injury (AKI). However, the mechanisms of the role of sTREM-1 for AKI remain unclear. It may be that topical inflammatory response of kidney, not just systemic inflammation, contributes to the elevated secretion of urine sTREM-1 in the process of sepsis-associated AKI. To further evaluate the role of sTREM-1 in this process, a larger-cohort multicenter study and the relevant basic research should be performed to reveal the diagnostic value and mechanism of sTREM-1 during the sepsis-associated AKI process. If successful, then urine sTREM-1 would be a good marker for sepsis and its associated AKI and could contribute to non-invasive diagnosis and monitoring in the clinical setting. Additionally, owing to the complexity of the pathogenesis of sepsis, it is necessary to combine some biomarkers to improve diagnostic performance in the diagnosis of sepsis-associated AKI rather than relying on a single marker.

Although the diagnostic and predictive values of plasma and urine neutrophil gelatinase-associated lipocalin (NGAL) and cystatin-C (Cys-C) for sepsis-associated acute kidney injury (AKI) are generally accepted as reliable [1, 2], the diagnostic value of urine soluble triggering receptor expressed on myeloid cells-1 (sTREM-1) is attracting more attention. In a recent article in *Critical Care*, Dai and colleagues [3] successfully collected plasma and urine and measured NGAL, Cys-C, and sTREM-1 and reported that both plasma and urine NGAL, Cys-C, and sTREM-1 levels were significantly increased at the time of diagnosis and 24 hours before AKI diagnosis. They once again proved urine sTREM-1 for sepsis-associated AKI diagnosis and confirmed the conclusion that was published by us in *Critical Care* in 2011 [4].

TREM-1 is a member of the immunoglobulin superfamily of receptors which is expressed on polymorphonuclear granulocytes and mature monocytes. It has been acknowledged that the levels of sTREM-1 in body fluid samples increased at the occurrence of infectious diseases, such as sepsis, pneumonia, septic arthritis, meningitis, peritonitis, and uterine cavity infection [5]. It was also reported that serum sTREM-1 could be used in the assessment of severity or even prognosis of sepsis [6, 7].

The advantages of sTREM-1 for the diagnosis of sepsis-associated AKI are the following: Firstly, the concentration of urine sTREM-1 stays high in sepsis; that is, the sTREM-1 concentration in the urine of patients with sepsis is 30 to 50 pg/ml, whereas the concentration of sTREM-1 in normal urine is almost undetectable. As the disease progresses or AKI appears, urine sTREM-1 elevated rapidly, suggesting poor prognosis. Secondly, urine sTREM-1 has great value for the early diagnosis of AKI, especially 24 and 48 hours prior to the AKI occurrence. The area under a receiver operating characteristic curve of urine sTREM-1 for AKI diagnosis was 0.7 to 0.9. Thirdly, dynamic change in the urine sTREM-1 also suggests the possibility of the AKI occurrence; that is, an increasing trend of sTREM-1 may predict the risk of AKI or even poor prognosis. Fourthly, it has been demonstrated that urine sTREM-1 is the risk factor for AKI occurrence in septic patients according to multivariate analysis logistic regression.

In a previous commentary on urine sTREM-1 assessment in diagnosing sepsis-related AKI, Derive and Gibot [8] raised a question: where does urinary sTREM-1 originate from? The alteration of both plasma and urine sTREM-1 concentration in the non-AKI patient with sepsis was mild but was very dramatic in the case of AKI. From both the plasma and urine concentrations of sTREM-1 during AKI, we think that secretion of sTREM-1 in plasma and urine may not be parallel. This paradoxical manifestation indicates that the topical inflammatory response of kidney could be involved in the AKI process, not just the systemic inflammatory response. Thus, sTREM-1 may be produced locally by the endothelial cells, tubular epithelial cells, or infiltrating inflammatory cells that are recruited during acute tubular necrosis. Consequently, sTREM-1 may be released into the blood circulation as a result of alteration in glomerular filtration barrier membrane pore size and charge. These possible mechanisms are summarized and illustrated in Fig. 1. Therefore, animal or cell experiments (or both) are necessary to confirm whether the use of TREM-1 inhibitors could block the process of sepsis-associated renal local inflammatory immune status.

**Fig. 1:**
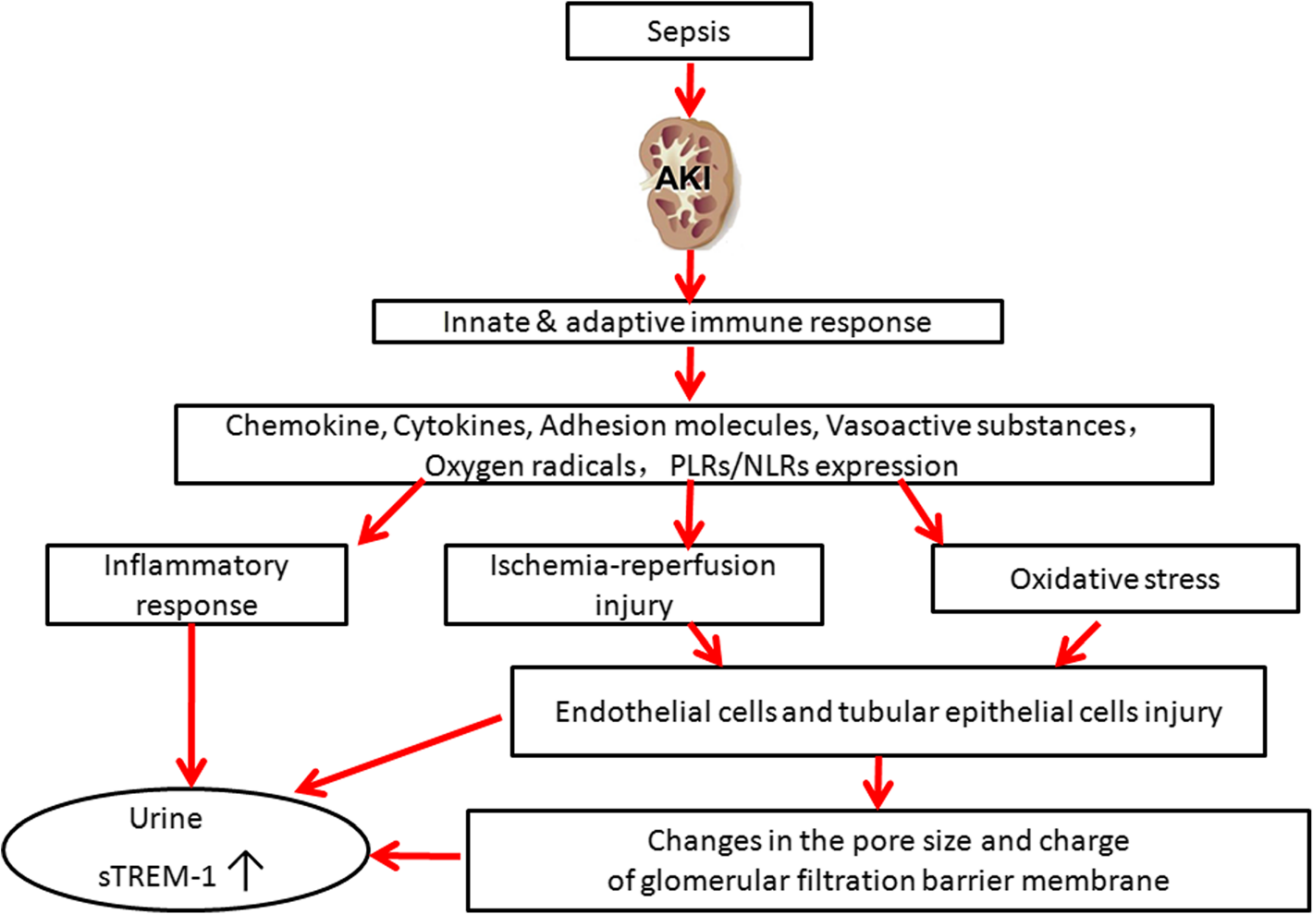
Schematic illustration of the possible mechanisms of urine sTREM-1 secretion in sepsis-associated AKI. *AKI* acute kidney injury, *NLR* Nod-like receptor, *TLR* Toll-like receptor, *sTREM*-*1* soluble triggering receptor expressed on myeloid cells-1

The increasingly successful example of the research of urinary biomarkers provides the opportunity for non-invasive detection and continuous monitoring of sepsis patients in the intensive care unit. It has been reported that sTREM-1 represented reliable diagnostic value for sepsis through meta-analysis [9, 10]. Of particular note, sTREM-1 could be secreted to and detected from urine during sepsis and its associated AKI; thus, we predict that urine sTREM-1 will be used for non-invasive and continuous monitoring for sepsis and its associated AKI. However, a large sample size and multicenter study are necessary to validate this potential biomarker. In this regard, since December 2012, a larger-cohort multicenter study joined by dozens of larger hospitals in the North of China has been in progress. From this large study, we expect the following: (1) the diagnostic value of urine sTREM-1 in sepsis and its associated AKI, especially for early diagnosis, will be confirmed; (2) the prognostic value of sTREM-1 in sepsis and its associated AKI will be assessed; (3) the correlation between sTREM-1 and classification of AKI proposed by Kidney Disease: Improving Global Outcomes (KDIGO) or the relationship between Acute Physiology and Chronic Health Evaluation II (APACHE II) and Sequential Organ Failure Assessment (SOFA) score will be evaluated; and (4) the influence of renal replacement therapy on the production and secretion of sTREM-1 will also be evaluated.

Each biomarker has its advantages and disadvantages. In some cases, it may be inappropriate to use certain biomarkers. For example, pre-existing kidney disease may interfere with the NGAL concentration by reabsorption through megalin-dependent endocytosis [11]. Septic patients with underlying diseases, such as cancer, or chronic kidney disease patients who are undergoing renal replacement therapy, or who are immunosuppressed or undergoing steroid treatment, may have false-negative results when these biomarkers are detected [12–14]. There seems to be no single indicator capable of confirming an AKI diagnosis. Therefore, a combination of biomarkers might be more valuable and precise in diagnosing sepsis-associated AKI. And it may be useful to develop a panel of biomarkers to improve the diagnostic efficacy of AKI. That is, some biomarkers—for example, combined NGAL, Cys-C, sTREM-1, or other biomarkers—should be taken together in diagnosing and assessing the prognosis of AKI. This could make it possible to increase the sensitivity and specificity of the diagnostic efficacy of AKI.

Urine sTREM-1 may become a non-invasive diagnostic and prognostic marker for sepsis and its associated AKI. Combined multiple biomarkers and their respective advantages may improve the diagnostic performance of sepsis and its associated AKI. A larger-cohort multicenter study and relevant basic research should be carried out to reveal its clinical significance and molecular mechanism.

## Abbreviations

AKI: Acute kidney injury
Cys-C: Cystatin C
NGAL: Neutrophil gelatinase-associated lipocalin
sTREM-1: Soluble triggering receptor expressed on myeloid cells-1
